# Coherent control of a donor-molecule electron spin qubit in silicon

**DOI:** 10.1038/s41467-021-23662-3

**Published:** 2021-06-03

**Authors:** Lukas Fricke, Samuel J. Hile, Ludwik Kranz, Yousun Chung, Yu He, Prasanna Pakkiam, Matthew G. House, Joris G. Keizer, Michelle Y. Simmons

**Affiliations:** 1 Centre of Excellence for Quantum Computation and Communication Technology, School of Physics, University of New South Wales, Sydney, Australia; 2 Present Address: Sussex Centre for Quantum Technologies, School of Mathematics and Physical Sciences, University of Sussex, Brighton, UK

**Keywords:** Spintronics, Qubits

## Abstract

Donor spins in silicon provide a promising material platform for large scale quantum computing. Excellent electron spin coherence times of ${T}_{2}^{* }=268$ *μ*s with fidelities of 99.9% have been demonstrated for isolated phosphorus donors in isotopically pure ^28^Si, where donors are local-area-implanted in a nanoscale MOS device. Despite robust single qubit gates, realising two-qubit exchange gates using this technique is challenging due to the statistical nature of the dopant implant and placement process. In parallel a precision scanning probe lithography route has been developed to place single donors and donor molecules on one atomic plane of silicon with high accuracy aligned to heavily phosphorus doped silicon in-plane gates. Recent results using this technique have demonstrated a fast (0.8 ns) two-qubit gate with two P donor molecules placed 13 nm apart in ^nat^Si. In this paper we demonstrate a single qubit gate with coherent oscillations of the electron spin on a P donor molecule in ^nat^Si patterned by scanning tunneling microscope (STM) lithography. The electron spin exhibits excellent coherence properties, with a ${T}_{2}$ decoherence time of 298 ± 30 *μ*s, and ${T}_{2}^{* }$ dephasing time of 295 ± 23 ns.

## Abstract (web summary)

Multi-donor molecules in Si provide a promising qubit platform, offering advantages over single donor qubits in terms of performance and fabrication. Here, the authors report a single qubit gate and long coherence times in a P donor molecule qubit in natural Si patterned by scanning tunneling microscope hydrogen lithography.

## Introduction

Atom qubits in silicon^1^ rely on using the potential well naturally formed by the donor atom nucleus to bind the electron spin at cryogenic temperatures allowing for reproducible qubits with a low gate density^2^. To date, electron spin qubits in ^nat^Si have demonstrated ${T}_{2}^{* }=55$ ns^3^ increasing to ${T}_{2}^{* }=268$ *μ*s in isotopically pure ^28^Si^4^ by addressing one of the phosphorus atoms statistically present in an ion implant region^5,6^. Atomic precision STM lithography allows us to pattern both individual single donor atoms, but also multi-donor molecules in silicon devices. Whilst single donors can trap up to two electrons, donor molecules can host multiple electrons, depending on number of donors present^7^. This ability to change the number of donors creates an enhanced tunability of the exchange interaction required for two-qubit gates^8^ and is known to reduce the impact of randomly placed charge impurities on both single and exchange coupled qubits^9^. Donor molecules have also been shown to produce longer spin relaxation times^10^ with an inbuilt addressability of spin resonance transitions allowing for lower error rates in multi-qubit control^11^.

In this paper we present coherent control of an electron spin on a multi-electron 1P–2P phosphorus donor molecule patterned by STM lithography in ^nat^Si with a Rabi frequency of 1.2 MHz and a ${T}_{2}^{* }$ dephasing time of 295 ns. Utilising the fifth electron spin on this molecule we observe a single narrow (7 MHz full width half maximum (FWHM)) electron spin resonance (ESR) transition, which we attribute to the reduced hyperfine interaction in our multi-electron system and potential nuclear spin pumping effects. Our results confirm that electron spin coherence times in ^nat^Si can be maintained in atomically engineered devices in silicon^12^, patterned close to heavily doped phosphorus leads and with multiple phosphorus nuclear spins in the qubit. These results are promising for precision engineered phosphorus in silicon architectures, where the transition to isotopically pure ^28^Si^13^ promises significant increases in coherence times^4^.

## Results and Discussion

**Fig. 1:**
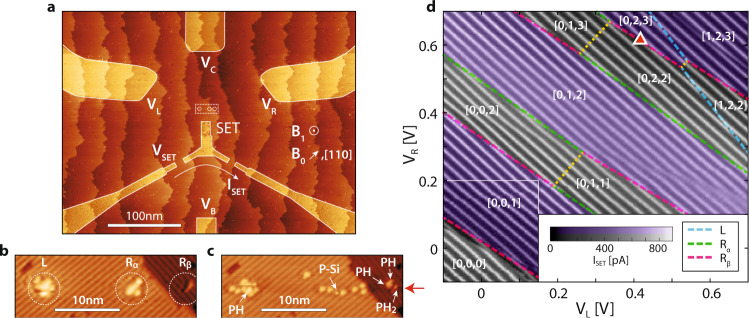
Charge stability diagram of a single P donor (*L*) and a 1P–2P molecule (***R***_***α***_ − ***R***_***β***_). **a** Overview STM micrograph of the device during STM lithography. Bright regions are exposed silicon which are subsequently dosed with phosphine to form conductive leads to the central qubit region (dotted white rectangle). **b** The central device region before dosing, with three sites (*L*, *R*_*α*_, *R*_*β*_) circled. **c** The same area following phosphine dosing and imaging. Here additional STM imaging of the surface in the fast (horizontal) scan direction has resulted in the tip un-intentionally creating additional dangling bonds along a scan line (position marked by the red arrow). In the final dosed image surface features containing phosphorus are marked whilst all unmarked bright spots are single dangling bonds incapable of incorporating P atoms. **d** Measured charge stability diagram of the device, showing *I*_SET_ as a function of *V*_L_ and *V*_R_ (*V*_C_ = 750 mV, *V*_B_ = 300 mV). Discontinuities represent charge state transitions on the three sites (blue*-L*, green*-R*_*α*_ or pink*-R*_*β*_ dashed lines). Inter-site charge transitions between *R*_*α*_ and *R*_*β*_ are highlighted in yellow. White numbering gives the electron occupation numbers [*L*, *R*_*α*_, *R*_*β*_]. Spin readout of Zeeman doublet states is only possible within the purple shaded areas. The operating point for the spin control experiments is given by the triangular red marker.

The donor qubit is fabricated using STM hydrogen lithography, where we employ an STM tip to selectively desorb hydrogen atoms from the silicon surface with subsequent dosing with phosphine gas and a high temperature incorporation anneal^14^. The central device structure after lithography is shown in Fig. 1a. Control structures with a metallic doping density are shown in the bright regions. Bias voltages *V*_L_, *V*_C_, *V*_R_, *V*_B_ applied to in-plane gates provide electrostatic control over the charge state, and a single-electron transistor (SET), which is biased at *V*_SET_ ≈ 300 *μ*V, permits single-shot charge detection^15^ by monitoring the drain current *I*_SET_. The SET also acts as an electron reservoir for the donor sites, patterned at a distance of 18.5 nm from the SET.

The dotted rectangular central device region is enlarged in Fig. 1b, c, which show the donor incorporation sites before and after phosphine dosing, respectively. By examining the height profiles we can identify all features in the STM micrographs (see Supplementary Note 1 for a full analysis). To the left of the SET, we observe one PH fragment in site *L* after dosing. To the right of the SET we observe two incorporation sites. The first site, *R*_*α*_ incorporated a single P atom, indicated by the P–Si heterodimer labelled surrounded by seven dangling bond sites. From previous experiments we know that a single dangling bond cannot absorb and incorporate P into the surface^16^. The second site, *R*_*β*_ absorbed one PH_2_ and two PH species, giving rise to two incorporated P atoms. Collectively, these two sites *R*_*α*_ and *R*_*β*_, separated by 8 nm, form a 1P–2P molecular state which can bind up to five electrons. Extrapolating from a previously obtained tunnel coupling of 4.3 GHz for a 2P–3P(3e) system, we expect the tunnel coupling for the fifth electron case in our molecular system to be in the hundreds of GHz taking into account the reduced separation between the quantum dots (16 nm → 8 nm), the additional electrons loaded onto the system (3e → 5e), and the lower number of P atoms comprising the quantum dots (2P–3P → 1P–2P)^7^.

Following lithography, dosing and annealing, 50 ± 5 nm of epitaxial silicon was grown at 250 ^∘^C and electron beam lithography used to pattern surface ohmic contacts and a microwave antenna for ESR control^11^. Using etched registration markers, we align the antenna at a lateral distance of 300 ± 50 nm from the buried donors to produce an oscillating magnetic field *B*_1_ perpendicular to the substrate at the donors’ position. Measurements were performed in a dilution refrigerator at an electron temperature of ~250 mK and under the application of a static magnetic field of *B*_0_ = 1.45 T along the [110] crystallographic axis, within the plane as indicated in Fig. 1a.

With a positive potential on the two centre gates (*V*_C_ = 750 mV, *V*_B_ = 300 mV), we vary gate voltages *V*_L_ and *V*_R_ to obtain the charge stability diagram of the SET and donor system, shown in Fig. 1d. From the charge stability diagram we observe breaks in the SET current due to charge transitions from the three donor sites (*L*, *R*_*α*_ and *R*_*β*_) to the SET with three distinct slopes (blue, green and pink). The difference in slope of these charge transition lines in the stability diagram equates to a unique relative lever arm, *α* of the left and right gate to each dot, confirming the presence of three different charge sites where phosphorus has been incorporated. We identify several accessible charge transitions within the achievable gate range, giving up to a total of six electrons in the system in the upper right corner. At more negative gate voltages (*V*_L_ < 0 and *V*_R_ < 0) the system is fully ionised, see Supplementary Note 2. Increasing the gate voltages lowers the electrochemical potential of the bound states, progressively adding electrons in the order *R*_*β*_, *R*_*β*_, *R*_*α*_, *R*_*α*_, *R*_*β*_, *L* (along the *V*_R_ = *V*_L_ line).

From charge stability diagrams using different combinations of gates we extract the ratio of the lever arms of each gate set to the donor dots. We then use these ratios, in combination with electrostatic finite element modelling, to triangulate^2^ and thus confirm the positions of the three donor dot sites *L*, *R*_*α*_ and *R*_*β*_ (see Supplementary Note 3). We also calculate the addition energies from the stability diagram, and confirm that the incorporation sites *R*_*α*_–*R*_*β*_ indeed consist of a 1P–2P molecular configuration, see Supplementary Note 4. We find that whilst each site has well-defined charge states there is strong capacitive coupling between them with a mutual charging energy ${E}_{{R}_{\alpha }-{R}_{\beta }} \sim 40$ meV. Moreover, we find that the spin parity is determined by the total number of electrons across both *R*_*α*_–*R*_*β*_ and not their individual electron number. For example, if *R*_*β*_ were an isolated quantum dot, spin readout^17,18^ should be possible when *R*_*β*_ is hosting 1 (odd) electron, e.g. in the [0,1,1] charge region. However, we observe that spin readout is only possible in the purple shaded regions in Fig. 1d where the total spin count of both *R*_*α*_ and *R*_*β*_ is odd. This result strongly suggests that the unpaired spin states on *R*_*α*_ and *R*_*β*_ form molecular singlet states, arising from the strong exchange coupling *J* between them. For spin readout we used a magnetic field of *B*_0_ = 2.5 T, allowing us to estimate that *J* > *gμB*_0_ ~ 70 GHz.

The ESR results we present next are obtained for the [*L*; *R*_*α*_; *R*_*β*_] = [0; 2; 3] five-electron charge state, at the position of the red triangle marker in Fig. 1d, where the first four electrons in the molecule occupy magnetically inactive singlet states. We note that we obtained ESR spectra for different electron occupations of the donor molecule with hyperfine values that are in agreement with tight binding calculations of the 1P–2P molecular state and this work will be published elsewhere.

**Fig. 2:**
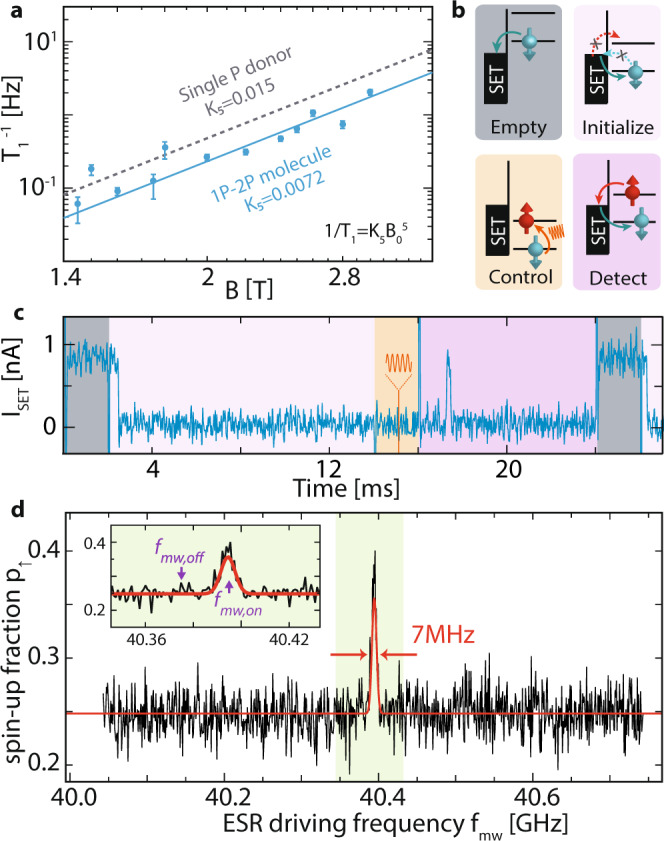
Spin relaxation and resonance spectrum of the 1P–2P molecule’s five-electron state. **a** Spin relaxation rate measurement of the five-electron state, as a function of magnetic field. Error bars indicate 95% confidence intervals. The known *T*_1_ time of a single electron bound to a single P donor is shown in grey. **b** Energy level diagrams for each stage of the pulse sequence used in the spin control experiments, showing the potentials of the Zeeman states relative to the SET Fermi energy. **c** Example readout trace of the SET current as a function of time, for a representative four stage pulse sequence incorporating an ESR control pulse. **d** Electron spin resonance spectrum of the five-electron state of the 1P–2P molecule at *B*_0_ = 1.45 T. The fitted peak width is 7 MHz. The inset displays a narrower range of frequency settings and marks the two frequencies *f*_mw,off_, *f*_mw,on_ used in the coherent driving experiments.

Spin-lattice relaxation in donor based qubits is known to be limited by acoustic-phonon mediated valley repopulation^19^, with a dependence of the relaxation rate *T*_1_ on the magnetic field *B*_0_ of the form ${T}_{1}^{-1}={K}_{5}{B}_{0}^{5}$. As shown in Fig. 2a, we find a spin-lattice relaxation time of *T*_1_ ≈ 16 s (at *B*_0_ = 1.45 T), and a proportional constant *K*_5_ = 0.0072 T^−5^ Hz for the 1P–2P molecule. We can compare these results to earlier measurements for a single P atom where *T*_1_ ≈ 6 s^17,18^ and *T*_1_ ≈ 15 s for a 2P quantum dot at *B*_0_ = 1.5 T^10^. Whilst the values are very similar we note that these earlier *T*_1_ measurements were recorded for the first electron on either the single donor or the 2P quantum dot. This is in contrast to the fifth electron on our molecular 1P–2P system. Since the fifth electron is most likely to be delocalised across the molecule, our results represent the first spin relaxation measurements of a molecular 1P–2P system and as such are distinct from previous results.

Since the long spin lifetime does not allow for fast initialisation by relaxation, we employ a four-level pulse sequence based on controlled tunnelling to and from the SET, which we show in Fig. 2b. The spin state is first emptied, leaving the molecule in a spin 0, even parity charge state (four electrons total). To initialise the spin, an odd parity electron is loaded deterministically spin-down by aligning the SET Fermi energy between the two Zeeman split spin $\frac{1}{2}$ states. This state is then isolated from the SET by electrostatically plunging far below the Fermi energy during the application of an ESR control pulse via the microwave antenna. Finally, the Zeeman split state is brought near the system’s Fermi energy, producing a detectable change in *I*_*SET*_ depending on the spin state. Each full sequence takes 24 ms, limited by the electron tunnel rate (~3 kHz) to and from the SET, with a single example time trace shown in Fig. 2c. We obtain statistics of the fraction of spin-up outcomes *p*_*↑*_ by repeating this pulse sequence.

The molecule’s ESR spectrum for the five-electron state is shown in Fig. 2d. The figure plots *p*_*↑*_ as a function of microwave frequency *f*_mw_ for an external field of *B*_0_ = 1.45 T, microwave power of *P*_*MW*_ ~18 dBm (before ~70 dB attenuation in the transmission line) and microwave pulse duration of 330 ns using a single tone at the given frequency. As shown in more detail in the inset which focuses on the centre region (the shaded area marks the frequency range of the inset), we only observe one resonance at a frequency of *f*_mw_ = 40.394 GHz. Superimposed is a fit to a single gaussian peak (red line) with a FWHM of *δf*_FWHM_ = 7 ± 1 MHz. The linewidth can be attributed to Overhauser fluctuations of the background spin bath of ^29^Si atoms in natural silicon^20^.

The existence of a single peak in the ESR spectrum of Fig. 2d has two potential explanations. Firstly, that the hyperfine coupling between the P donor nuclear spins and the bound electron spin is small. It has been shown that the hyperfine coupling of the third electron on a 2P quantum dot is reduced by a factor of ≈10 compared to a single electron on a single P atom^21^. Here the outer electron spin is screened from the positively charged donor cores by the spin-paired electrons of the inner orbitals and results in a lateral spread of the outer electron wavefunction and a reduced charge density and hyperfine interaction at the central nuclear sites^11^. For the fifth electron on our 1P–2P molecular state an even larger reduction in hyperfine coupling might be expected due to the presence of four electrons shielding the inner core. Depending on the exact geometrical donor configuration^21^, the hyperfine coupling in donor molecules with one electron typically range between tens to hundreds of MHz. With five electrons on our 1P–2P molecule the hyperfine coupling is likely to be no more than a few MHz. This value is comparable to the Overhauser field broadened ESR linewidth in ^nat^Si and prevents us from resolving any splitting of the peak experimentally.

Whilst the observation of a single ESR line can be explained by the reduced hyperfine splitting from the increased lateral extent of the wavefunction of the fifth electron, we cannot rule out the alternate explanation that only one nuclear spin configuration is observed in our measurement. This could arise from a nuclear spin pumping effect driven by the repeated loading and excitation sequence^22^, effectively forcing the phosphorus nuclei into a fixed spin configuration during the measurement.

We measure the electron *g*-factor by varying the external magnetic field *B*_0_ and observing the resonance condition *hf*_mw_ = *gμ*_B_*B*_0_ (with *h* Planck’s constant and *μ*_B_ = 57.9 *μ*eV/T the Bohr Magneton). We find *g* = 1.99 ± 0.02 in agreement with the accepted value for phosphorus donors in silicon^23^, with an uncertainty limited by the calibration of our superconducting magnet, see Supplementary Note 5.

In the coherent driving experiments, we employ an interleaved measurement method where we constantly toggle the driving frequency between the two frequencies marked in Fig. 2d, *f*_mw,on_ = 40.394 GHz (on resonance) and *f*_mw,off_ = *f*_mw,on_ − 20 MHz (off resonance). In this way we constantly measure a background signal when exciting off-resonance, which allows us to display the results as Δ*p*_*↑*_ = *p*_*↑*_(*f*_mw,on_) − *p*_*↑*_(*f*_mw,off_), independent of classical effects such as heating, charge noise or bias voltage drift, which can alter the background spin-up fraction over time. Both *p*_*↑*_(*f*_mw,on_) and *p*_*↑*_(*f*_mw,off_) are obtained using a single-shot readout protocol shown in Fig. 2c.

**Fig. 3:**
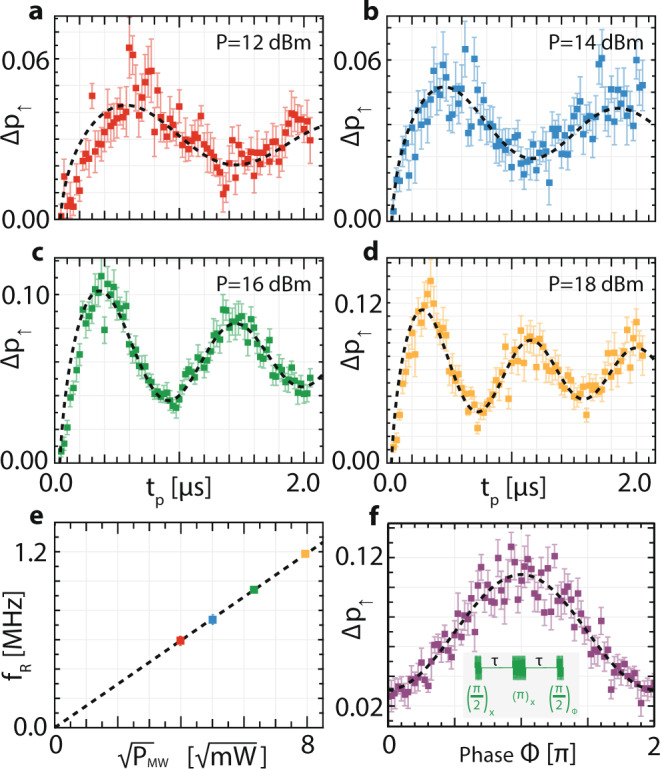
Coherent spin control of the molecular electronic state. **a**–**d** Spin-up difference signals Δ*p*_*↑*_ for four different mircowave power values as a function of pulse duration, showing clear Rabi oscillations. We note that Δ*p*_*↑*_ does not reach 1 since the echo sequences are performed by driving a single frequency tone while the fluctuating Overhauser field in natural silicon detunes the instantaneous resonace from the driven frequency as has been observed previously^20^. Error bars indicate ±1*σ*, and black dashed lines indicate fits to a power-law decay. For the highest driving strength in **d** we obtain a Rabi frequency of *f*_R_ = 1.18 MHz. **e** The extracted Rabi frequencies as a function of microwave amplitude ${B}_{1} \sim \sqrt{{P}_{{\mathrm{MW}}}}$ show a linear dependence. **f** Phase control, demonstrated by a spin echo sequence in which the initial and final $\frac{\pi }{2}$ rotations differ in phase by *ϕ*, producing a sinusoidal variation on the recovered spin state. Experimental parameters are *P*_MW_ = 14 dBm, *t*_*π*_ = 840 ns and *τ* = 50 ns, and the pulse sequence is indicated in the inset.

Coherent control is achieved by varying the pulse duration for fixed microwave power and frequency and extracting the spin-up fraction using the single-shot spin readout method outlined above. The observed Rabi oscillations in Fig. 3a–d for different settings of microwave power, exhibit non-exponential decay envelopes due to the fluctuating Overhauser field of ^29^Si nuclear spins. The instantaneous Overhauser field component parallel to *B*_0_ contributes an unknown detuning relative to the expected electron resonance condition, causing the spin to accumulate phase at an unknown rate. This detuning also modifies the rate of driven spin rotation during an applied ESR pulse, as greater detuning tilts the effective rotation axis out of the plane perpendicular to *B*_0_.

For this experiment we are in the so-called weak-driving regime, where the Rabi frequency (≤1.2 MHz) is less than the width of the Overhauser field distribution (7 MHz)^24,25^. In this regime, the decay profile for times beyond the first oscillation period is well described by a power law envelope together with a phase shift of $\frac{\pi}{4}$. From fits to the formula:

1 $${{\Delta }}{p}_{\uparrow }({t}_{p})=\frac{a}{\sqrt{{t}_{p}}}\cos \left(2\pi {f}_{\text{R}}{t}_{p}+\frac{\pi}{4}\right)+c,$$

with *a*, *c*, *f*_R_ as free parameters, indicated by dashed black lines in Fig. 3a–d, we derive the ensemble average Rabi frequency *f*_R_ for each power setting. Parameters *a* and *c* describe the visibility of the oscillations, and reflect the variation in Overhauser field values across the time ensemble of single-shot measurements together with state preparation and readout errors.

In total ≈11,400,000 single-shot traces are represented in these graphs, from which we extract the expected linear dependence between the amplitude of the driving field ($\sqrt{{P}_{{\mathrm{MW}}}}$) and the Rabi frequency as shown in Fig. 3e. At the highest power setting we see a Rabi frequency of *f*_R_ = 1.18 ± 0.02 MHz, and on average an initial *π* rotation within about 376 ns.

The Overhauser field variations can be partially overcome using spin echo techniques. In Fig. 3f we demonstrate a spin echo sequence (as indicated in the inset) consisting of a $\frac{\pi }{2}$ rotation from the initial spin-down state, a short delay of time *τ* = 50 ns, a refocusing *π* pulse which serves to correct for any unknown phase accumulated throughout the sequence, and following a second delay of *τ*, a final $\frac{\pi }{2}$ rotation of varying phase *ϕ* relative to the first two pulses. For *ϕ* = 0, the three pulses together result in a total rotation of angle $\frac{\pi }{2}+\pi +\frac{\pi }{2}=2\pi$ about the X-axis and the final spin outcome is spin-down. For *ϕ* = *π*, the final pulse rotates the state in the opposite direction, leaving the final state as spin-up. For intermediate values of the phase angle the final rotation leaves the spin state as a superposition. The data is fit to a sinusoid:

2 $${{\Delta }}{p}_{\uparrow }({{\Phi }})=c-a\cos ({{\Phi }}),$$

superimposed as a black dashed line. The result in Fig. 3f demonstrates two axis control over the state of the electron spin, enabling any single qubit unitary transformation by a combination of X (*ϕ* = 0) and Y ($\phi =\frac{\pi }{2}$) axis rotations.

**Fig. 4:**
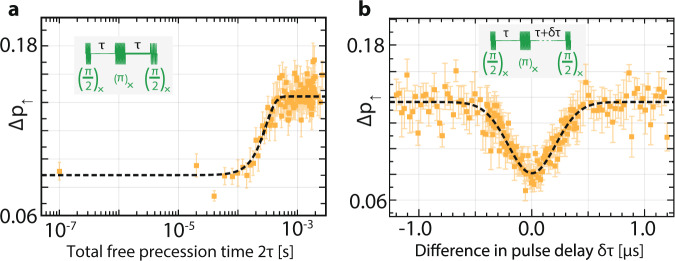
Decoherence and dephasing times of the molecular five-electron spin state. **a** Hahn spin echo measurement of the five-electron state, plotting the measured spin-up fraction Δ*p*_*↑*_ as a function of total free precession time 2*τ*. **b** Spin echo envelope measurement with a Ramsey-type pulse sequence. The initial free precession time *τ* = 3 *μ*s is fixed, and we plot Δ*p*_*↑*_ as a function of the relative delay of the final projection pulse *δτ*. Error bars indicate ±1*σ* on the spin-up fraction.

We also study the coherence properties of our spin system by applying compound pulse sequences. We perform a Hahn echo experiment, in which we initialise the electron spin-down, then apply $\frac{\pi}{2}, {\pi}$ and $\frac{\pi}{2}$ pulses about the X-axis, separated by time *τ* as shown in the inset to Fig. 4a before spin readout. Operating at *P*_MW_ = 18 dBm, we set the *π* time in the Hahn echo experiment to *t*_*π*_ = 376 ns and vary the free precession time 2*τ* in Fig. 4a. For short times, we recover the spin-down state, and for very long evolution times, the spin state becomes completely randomised due to complete dephasing caused by uncontrolled fluctuations in the spin bath. The transition between the two regimes is described by a compressed exponential^26^ of the form:

3 $${{\Delta }}{p}_{\uparrow }(2\tau)=c-a\exp \left(-{\left(\frac{2\tau}{{T}_{2}}\right)}^{n}\right),$$

shown as a black dashed line. While fit parameters *c* and *a* define the visibility, the coherence properties are captured by an exponent *n* = 2.6 ± 1 and the Hahn echo decoherence time *T*_2_ = 298 ± 30 *μ*s which are both remarkably close to values measured for phosphorus in bulk ^nat^Si^26^. This result indicates that decoherence is mainly due to a slowly fluctuating bath of ^29^Si nuclear spins^27^.

Changing the timing of the last projection pulse by introducing a deviation of *δτ* from a fixed free evolution time *τ* = 3 *μ*s (shown in the inset to Fig. 4b results in a Ramsey-type spin echo envelope experiment. Phase coherence with the Larmor precession due to *B*_0_ is lost if the instantaneous Overhauser detuning is comparable to $\frac{1}{\delta \tau }$. We sample this spin dephasing across the time ensemble of single shot measurements to determine the pure dephasing time ${T}_{2}^{* }$. Fitting the data in Fig. 4b to a gaussian distribution with standard deviation *σ*, we obtain ${T}_{2}^{* }=\sqrt{2}\sigma =295\pm 23$ ns. This value is about five times longer than observed in an ion implemented device measuring an individual P phosphorus atom^20^ in natural silicon. We note however that electron spin dephasing can be influenced by interaction with the nuclear spin bath, which depends on the time dynamics of the hyperfine interaction under a specific experimental pulse sequence^28^. Accurately modelling the decoherence time would require a full simulation of the multi-electron few-donor wavefunction^29^, along with a method such as correlated cluster expansion^30^ to account for non-classical memory effects in the interacting ^29^Si atoms. However, we can qualitatively attribute the extended dephasing time ${T}_{2}^{* }$ to the reduced electrostatic confinement and thereby more extended wave function of our five-electron molecular state. This results in a weaker coupling to a larger ensemble of nuclear spins, producing a narrower distribution of Overhauser field values. At the same time, our molecular confining potential does not significantly impact the concentration of ^29^Si nuclear spins or the timescale of their flip-flop dynamics, leaving the *T*_2_ decoherence time observed via Hahn echo comparable to that seen for a single phosphorus in bulk ^nat^Si^26^.

In conclusion, we have demonstrated coherent control of an electron spin bound to a 1P–2P donor molecule in isotopically natural silicon. We found that the inhomogeneous dephasing time ${T}_{2}^{* }$ is five times longer than that observed for a similar experiment performed on a single P donor. Along with the intrinsic addressability of donor molecules, and their advantages for achieving controllable inter-qubit exchange coupling, we confirm that multi-electron states bound to donor molecules provide a long-lived coherent spin resource for quantum computing. This work confirms that the presence of numerous dopant atoms within the planar conductive leads of a donor-defined readout SET, along with multiple nuclei within the donor qubit, does not limit its coherence.

## Methods

### Device fabrication

The STM hydrogen lithography was done at a pressure below 1*e*^−11^ mbar with an Omicron Variable Temperature instrument. A chemically cleaned Si(001) wafer was passivated in a beam of atomic hydrogen after a 1100 ^∘^C reconstruction anneal. The hydrogen mask was selectively removed by scanning with a tip voltage of 3–6 V and current setpoint of 1–10 nA. After lithography, phosphine dosing and imaging, the wafer was heated to 350 ^∘^C before a 55 nm layer of epitaxial silicon was grown at a rate of 0.15 nm/min to encapsulate the incorporated donors. The donor layer was electrically contacted by depositing aluminium over contact vias produced by reactive ion etching. The contact structures, as well as the microwave antenna were all defined by electron beam lithography using a PMMA mask.

### Cryogenic measurements

Spin resonance measurements were performed at 50 mK in a dilution refrigerator. A superconducting solenoid magnet provided the external magnetic field. DC voltage offsets were generated by Yokogawa 7651 and Stanford Research Systems SIM928 voltage sources. Time-varying voltage pulses were generated by a National Instruments USB6363 DAC/ADC device and added to the static offsets via resistive voltage dividers. The combined gate control signals were filtered by two-stage lumped element RC filters inside the dilution fridge with a low-pass cutoff of 150 kHz. The microwave signals were supplied to the on-chip antenna from a Keysight E8267D vector signal generator (with phase and pulse modulation signals supplied by a Tektronix 5014C arbitrary waveform generator) via a lossy stainless steel coaxial cable running from room temperature to 50 mK. The SET current readout signal was amplified by a Femto DLPCA200 transimpedance amplifier and then electrically decoupled and filtered by a Stanford Research Systems SIM910 JFET isolation amplifier and SIM965 Bessel filter before being digitised by the National Instruments USB6363 DAC/ADC.

## Supplementary information

Supplementary Information

## Data Availability

The data that support the findings of this study are available from the corresponding author upon reasonable request.
