# Supplementary material for: Coherent control of a donor-molecule electron spin qubit in silicon
Source: Nat Commun. 2021 Jun 3;12:3323. doi: 10.1038/s41467-021-23662-3 (PMC8175606; doi:10.1038/s41467-021-23662-3)
Supplement: Supplementary file 1 — Supplementary Information [file 41467_2021_23662_MOESM1_ESM.pdf]

Supplementary Information

Coherent control of a donor-molecule electron spin qubit in silicon

Lukas Fricke, Samuel J Hile, Ludwik Kranz, Yousun Chung, Yu He, Prasanna Pakkiam, Matthew G House, Joris G Keizer, and Michelle Y Simmons

I. SUPPLEMENTARY NOTE 1: STM ANALYSIS

By examining the height profiles of the STM micrographs we can identify all features on the surface before the incorporation anneal, see Supplementary Fig. **1**. We conclude that site R_α consists of a single P–Si heterodimer, incorporated during scanning, and site R_β contains two PH species and one PH_2. After the incorporation anneal site R_α is certain to yield a 1P since the P is already incorporated into the Si surface. Site R_β will most likely yield 2P atoms with the first PH shedding a H that bonds with the single DB on the same dimer row and the second PH shedding a H atom which subsequently combines with the PH_2 to from a desorbed PH_3 molecule[1,2], allowing the remaining 2 P atoms to incorporate. We note here that the subsequent incorporation of phosphorus atoms into the silicon is a chemical process that, to an extent, is probabilistic in nature. Nevertheless, from previous studies of phosphorus incorporation in small regions[3–9], we estimate that we can determine the number of incorporated P with a ∼80% accuracy.

II. SUPPLEMENTARY NOTE 2: EXTENDED CHARGE STABILITY DIAGRAM

In order to confirm the absolute electron number on our phosphorus dots a charge stability diagram taken at larger negative gate voltages than presented in main text Fig 1d is recorded. Supplementary Fig. **2**a, shows an extended stability diagram of V_L *vs.* V_R taken with the same ($V_C = 750\,\mathrm{mV}$ and $V_B = 300\,\mathrm{mV}$) gate voltages applied, whilst Supplementary Fig. **2**b was taken with no bias applied ($V_C = 0\,\mathrm{mV}$ and $V_B = 0\,\mathrm{mV}$), opens up a larger region of gate space. Beside the 6 charge transitions of our donor dots in Fig. **2**a we see no additional charge transitions of these dots at more negative gate bias. The near horizontal lines in Fig. **2**b correspond, given their slope, to unintentional charge traps away from the inner region of our device and in close proximity to the right gate. The reduction in SET current around $V_L = -0.35\mathrm{V}$ and $V_R = 0\mathrm{V}$ is due to an elastic co-tunnelling process unique to triple dot systems[10] known as quantum cellular automata (QCA) and is not a charge transition.

III. SUPPLEMENTARY NOTE 3: ELECTROSTATIC TRIANGULATION

To confirm the positions of sites L, R_α, and R_β, we employ a simple electrostatic triangulation method[11] using the charge-transition lines as observed in a set of charge stability diagrams. Each of these charge stability diagrams in Supplementary Fig. **3** involves using a different pair of gate electrodes, with the other gates set to fixed biases to obtain a planar slice through the multi-dimensional space of gate voltage combinations. As a result, the observed slope of each dot's charge transition lines, and their apparent separation, varies. The geometry of the STM device, see **4**a, is imported into an electrostatic finite-element model in which each of the STM patterned metallic phosphorus leads and the SET are represented as perfect electrical conductors with the lithographically patterned STM dimensions and assuming a vertical thickness due to the Bohr radius of 2 nm. The electrostatic lever arm of an electrode to an arbitrary point-like dot is given by $\alpha = -\Delta U/(e\Delta V)$, with ΔU the change in the dot's potential upon a change in gate voltage ΔV[12]. The slope of the charge transition line of a given dot in each charge stability diagram gives the ratio, γ, of lever arms of the gate combination with respect to each dot. This ratio is then associated with a region in the electrostatic model as the ratio of lever arms of two gates upon a dot at a given point and is equivalent to the ratio of electrostatic potentials at the given point. The resulting triangulation, shown as the shaded regions in Supplementary Fig. **4**b-c, shows that the spatial position of the dots identifies the lithographic location of the patterned dots.

IV. SUPPLEMENTARY NOTE 4: ADDITION ENERGIES

The addition energy can be extracted from a charge stability diagram[7,10] once the lever arm, α is known allowing us to convert the voltage difference, ΔV, as measured in the charge stability diagram to energy, see Supplementary Fig. **5**. The addition energy is given by: $E_{add} = \alpha\Delta V$. In this work we have extracted the left gate lever arms to the 3 quantum dots to be $\alpha_L = 0.051, \alpha_{R\alpha} = 0.033, \alpha_{R\beta} = 0.079$ from spin tail measurements. From the charge stability diagrams in Supplementary Fig. **5**, we can therefore extract ΔV to calculate the addition energies.

The resulting addition energies obtained are compared to the previously calculated addition energies for a 2P quantum dot as published in literature[7,10] and reproduced here as

Supplementary Fig. **6**, which shows the addition energies vs. transition number. For site R_β the addition energies of [1e→2e; 2e→3e] are (93±20 meV; 86±17 meV) respectively, where we find close agreement with the calculated addition energies for a 2P system. For site R_α we only observe two charge transitions with an addition energy [1e→2e] = 41±8 meV consistent with a single phosphorus atom which cannot bind more than 2 electrons[7,13]. This is supported by the STM micrograph which unambiguously shows that site R_α can only consist of a 1P.

V. SUPPLEMENTARY NOTE 5: *G*-FACTOR

In Supplementary Fig. **7**, we display the measured spin resonance peak position over a range of external magnetic field values. We find a Landé g-factor of $g = 1.99 \pm 0.02$ with the uncertainty limited by the calibration of our superconducting magnet.

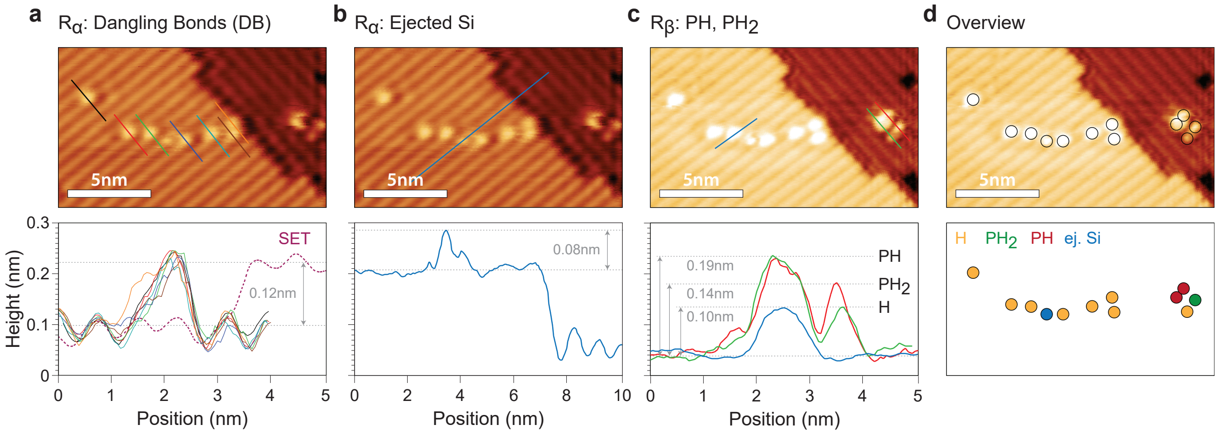

Supplementary Fig. 1. **STM image analysis of sites R_α and R_β before the incorporation anneal and Si encapsulation.** All images show the filled state (-1.7 V). **a)** Identification of single dangling bonds (DB) on the surface. The height of seven of the features (0.12 nm) matches the height of single dangling bonds reported in literature[4,14,15]. We also show for comparison the height of the intentionally depassivated SET region on the same surface (shown in pink). b) The height of the smaller feature in site R_α (0.08 nm) matches the height of a P–Si heterodimer[3] indicating that a single phosphorus atom has been incorporated at this site during the scanning. **c)** The height of the features in site R_β matches the height of 2× PH fragments (0.19nm), 1× PH_2 fragment, and a single dangling bond (0.10 nm). The height profile of a previous positively identified single dangling bond (DB, blue line) is shown for comparison. These height profiles provide clear identification of the species on the surface and correspond well with values previously reported in literature[3,4,16]. **d)** The final image of the sites R_α and R_β before the incorporation anneal with the different atomic species labelled and their position marked with coloured circles for clarity. From a detailed understanding of the chemical reactions that occur during the incorporation anneal[2,13] we conclude that site R_α, where we observe a single P–Si heterodimer, is certain to yield a single phosphorus atom upon the incorporation anneal since the P is already incorporated into the Si surface at this point. Site R_β will yield two phosphorus atoms with the first PH shedding a H atom that bonds with the single DB on the same dimer row and the second PH shedding a H that combines with the PH_2 to form a PH_3 molecule which desorbs from the surface allowing the remaining 2 P atoms to incorporate on annealing[1,2].

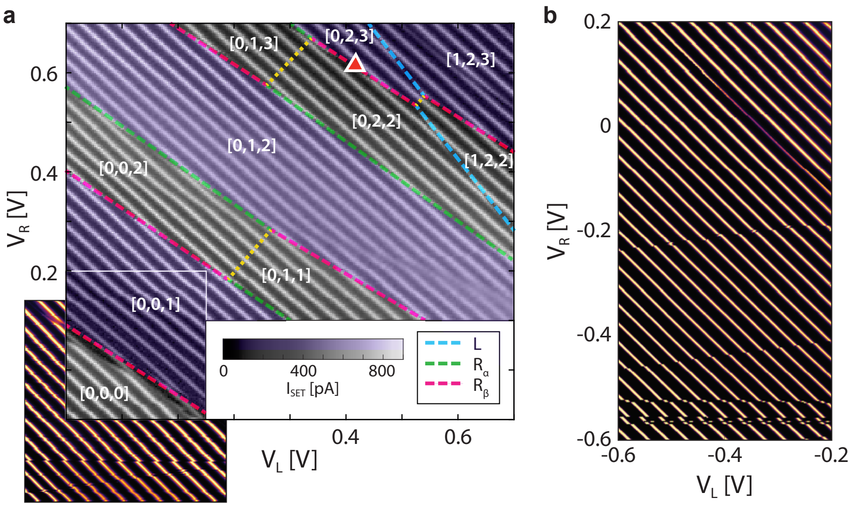

Supplementary Fig. 2. **Extended charge stability diagram** taken with **a)** $V_C = 750$mV and $V_B = 300$mV and **b)** with $V_C = 0$V and $V_B = 0$V. In this voltage range no additional charge transitions of the phosphorus donor dots are observed. We do however observe additional charge transitions at the highest negative biases which we attribute to unintentional charge traps due to their near horizontal slope in the stability diagram. In these plots, the colour brightness corresponds linearly to the measured SET current. The reduction in SET current around $V_L = -0.35$V and $V_R = 0$V is due to an elastic co-tunnelling process unique to triple dot systems[10] known as quantum cellular automata (QCA) and is not a charge transition.

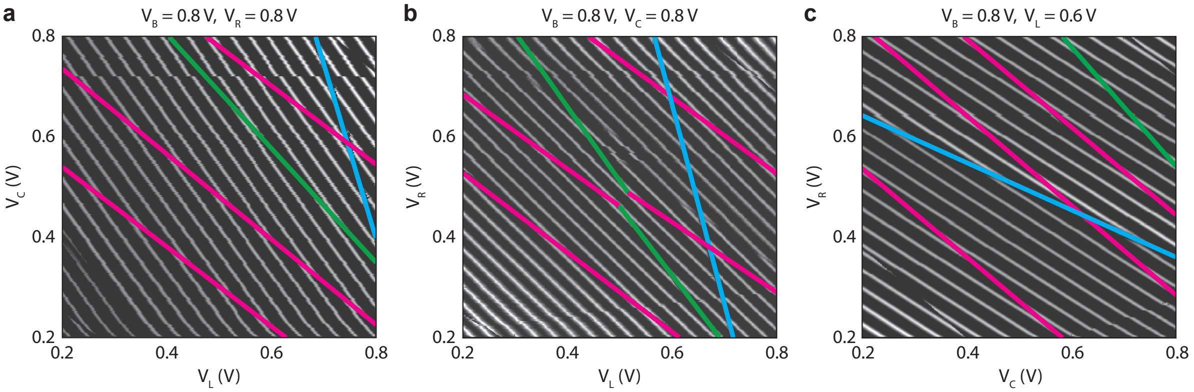

Supplementary Fig. 3. **Charge stability diagrams with different gate combinations. a)** V_L vs. V_C, **b)** V_L vs. V_R, and**c)** V_C vs. V_R. All stability diagrams show charge transitions from the L, R_α and R_β dots with three distinct slopes (blue, green, purple). From these charge stability diagrams the ratio of lever arms of the gates to the quantum dots ($\gamma = \alpha_{1-2} = \Delta V_1/\Delta v_2$) can be extracted. In these plots, the colour brightness corresponds linearly to the measured SET current.

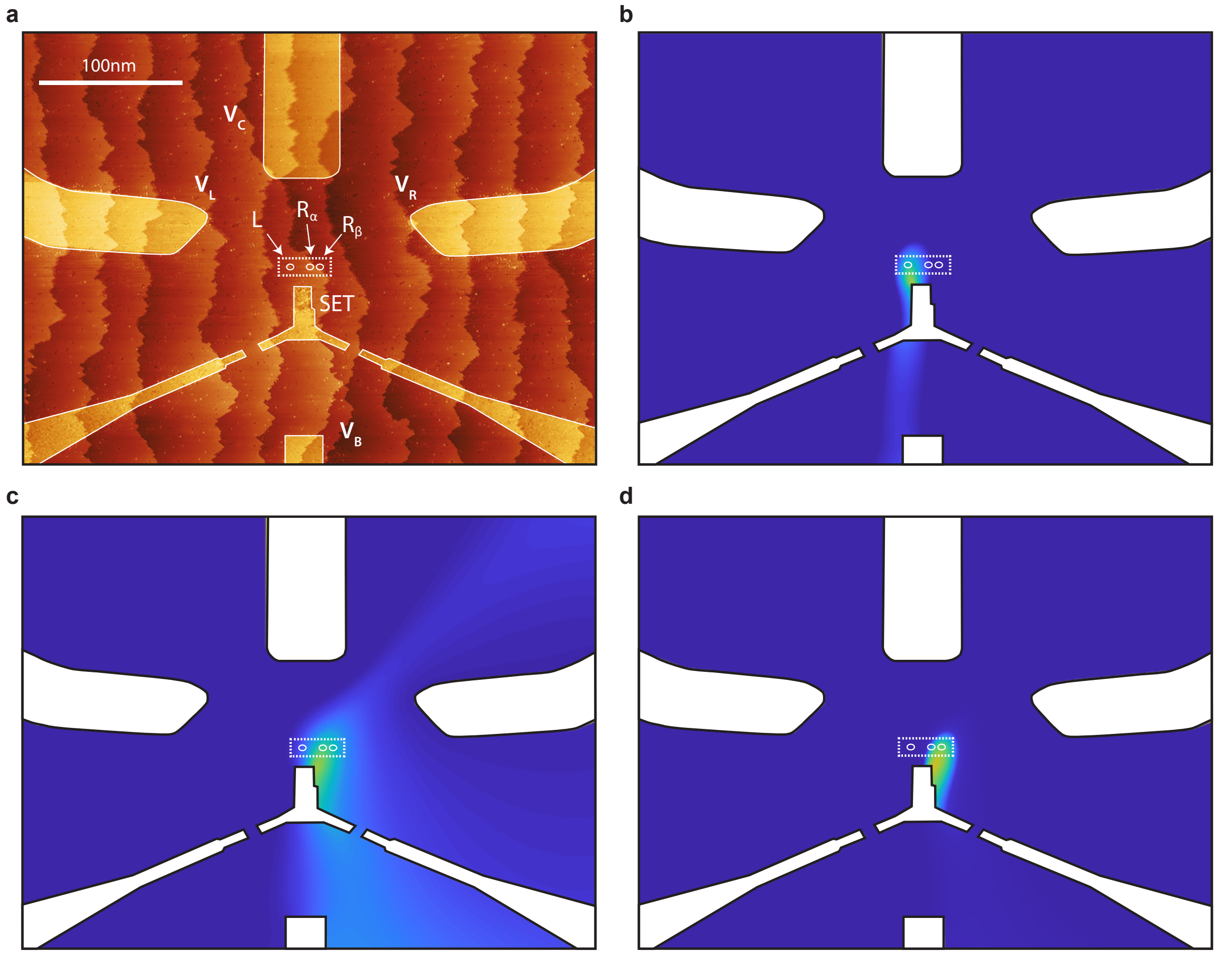

Supplementary Fig. 4. **Electrostatic modelling confirms the location of the phosphorus incorporation sites. a)** STM micrograph from which the exact gate geometry is extracted. Using different gate combinations (V_L–V_C, V_R–V_C, V_L–V_R) and the slopes of the charge transitions in the corresponding charge stability diagrams, the position of the incorporation sites is triangulated for site b) L, c) R_α, and d) R_β. The coloured shaded region corresponds to a Gaussian distribution of the measured slope based on 10% uncertainty. The resulting triangulation, where the shaded regions overlap, shows that the spatial position of the dots identifies the lithographic locations of the patterned dots highlighted by the dashed circles.

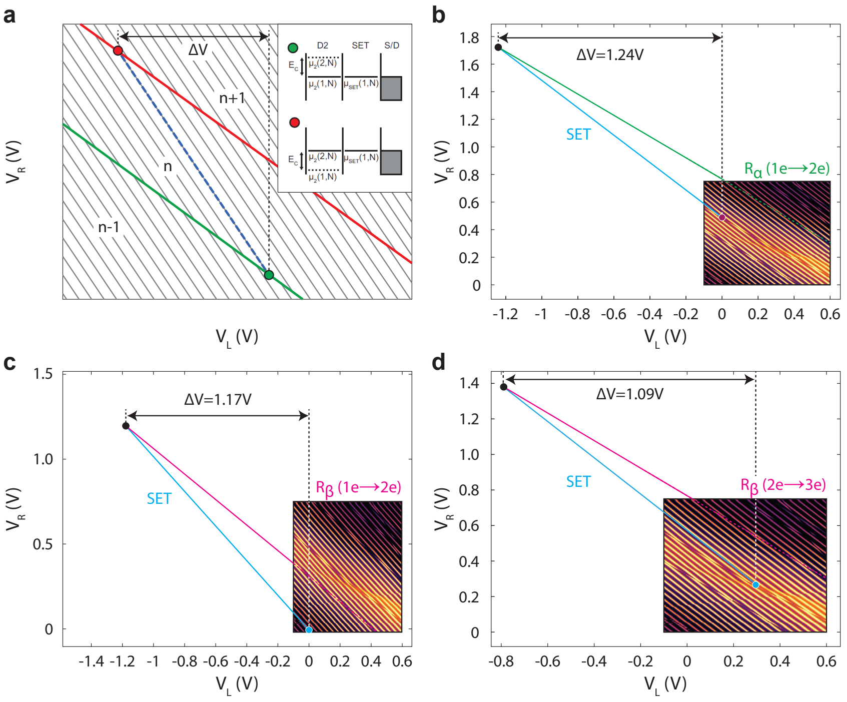

Supplementary Fig. 5. **Determining the addition energy from charge stability diagrams. a)** Schematic representation of the procedure used to extract the addition energy, E_{add} of the $n \rightarrow (n+1)$ transition. Inset: energy level diagrams show that at the green and red circles the n and $(n+1)$ electrochemical potentials are aligned with the SET electrochemical potential. **b,c,d)** Extracting ΔV for the R_α(1e⟶2e), R_β(1e⟶2e), R_β(2e⟶3e) transitions.

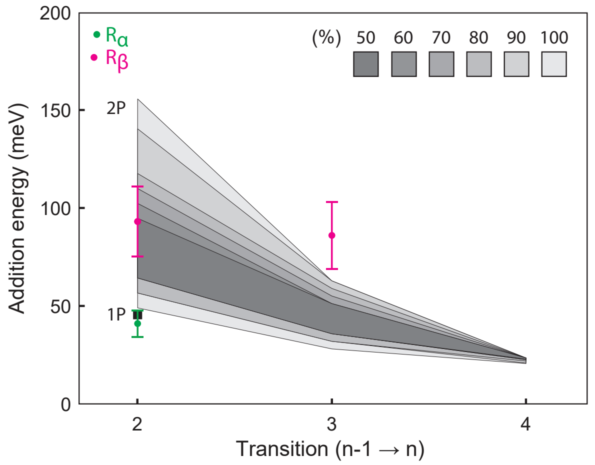

Supplementary Fig. 6. Calculated addition energies for a 1P and 2P quantum dot as a function of charge transition (reproduced from[10]). All possible donor configurations within a volume that includes 1 nm in all directions of the lithographically defined dot are considered in the model to account for donor diffusion, resulting in the percentile likelihood (%) bands. The experimentally obtained addition energies of the quantum dots at site R_α (green) and R_β (pink) are marked in the graph.

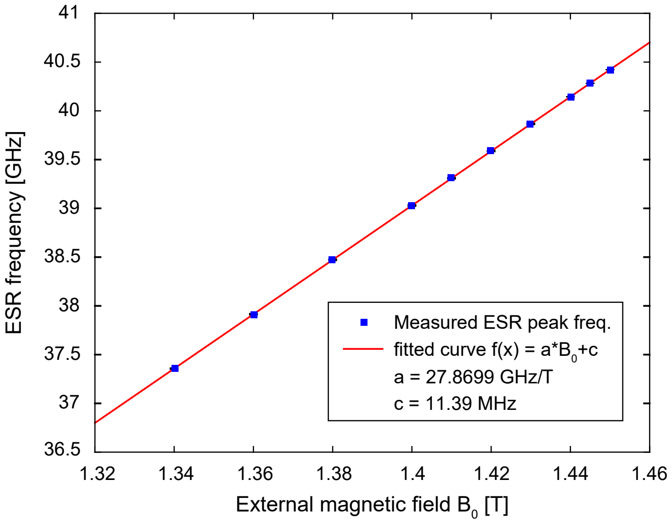

Supplementary Fig. 7. Centre ESR peak frequency as function of the external magnetic field B_0. The experimentally obtained centre ESR peak frequency (blue points) is fitted (red) to a linear function. We extract a Landé g-factor of $g = 1.99 \pm 0.02$ with the uncertainty limited by the calibration of our superconducting magnet.

REFERENCES

[1]Wilson, H. F. *et al.* Thermal dissociation and desorption of PH_3 on Si(001): A reinterpretation of spectroscopic data. *Physical Review B - Condensed Matter and Materials Physics* **74**, 195310 (2006).

[2]Warschkow, O. *et al.* Reaction paths of phosphine dissociation on silicon (001). *Journal of Chemical Physics* **144**, 014705 (2016).

[3]Hallam, T. *The use and removal of a hydrogen resist on the Si(001) surface for P-in-Si device fabrication.* Ph.D. thesis, University of New South Wales (2006).

[4]Fuechsle, M. *Precision Few-Electron Silicon Quantum Dots.* Ph.D. thesis, University of New South Wales (2011).

[5]Fuechsle, M. *et al.* Spectroscopy of few-electron single-crystal silicon quantum dots. *Nature Nanotechnology* **5**, 502 (2010).

[6]Büch, H., Mahapatra, S., Rahman, R., Morello, A. & Simmons, M. Y. Spin readout and addressability of phosphorus-donor clusters in silicon. *Nature Communications* **4**, 2017 (2013).

[7]Weber, B. *et al.* Spin blockade and exchange in Coulomb-confined silicon double quantum dots. *Nature Nanotechnology* **9**, 430–435 (2014).

[8]Broome, M. A. *et al.* High-Fidelity Single-Shot Singlet-Triplet Readout of Precision-Placed Donors in Silicon. *Physical Review Letters* 046802 (2017).

[9]Hile, S. J. *et al.* Addressable electron spin resonance using donors and donor molecules in silicon. *Science Advances* **4**, eaaq1459 (2018).

[10]Watson, T. *Multi-qubit architectures for donor-based silicon quantum computing.* Ph.D. thesis, University of New South Wales (2015).

[11]Pakkiam, P., House, M. G., Koch, M. & Simmons, M. Y. Characterization of a Scalable Donor-Based Singlet-Triplet Qubit Architecture in Silicon. *Nano Letters* **18**, 4081–4085 (2018).

[12]Van der Wiel, W. G. *et al.* Electron transport through double quantum dots (2003). 0205350.

[13]Fuechsle, M. *et al.* A single-atom transistor. *Nature Nanotechnology* **7**, 242–246 (2012).

[14]Wang, Y., Bronikowski, M. J. & Hamers, R. J. Direct dimer-by-dimer identification of clean and monohydride dimers on the Si(001) surface by scanning tunneling microscopy.

Journal of Vacuum Science & Technology A: Vacuum, Surfaces, and Films **12**, 2051–2057 (1994).

[15] Lyding, J. W., Shen, T. C., Hubacek, J. S., Tucker, J. R. & Abeln, G. C. Nanoscale patterning and oxidation of H-passivated Si(100)-2×1 surfaces with an ultrahigh vacuum scanning tunneling microscope. *Applied Physics Letters* **64**, 2010–2012 (1994).

[16] Schofield, S. R. *et al.* Atomically precise placement of single dopants in Si. *Physical Review Letters* **91**, 136104 (2003).
